# RNA-seq analysis of the salivary glands and midgut of the Argasid tick *Ornithodoros rostratus*

**DOI:** 10.1038/s41598-019-42899-z

**Published:** 2019-05-01

**Authors:** Ricardo N. Araujo, Naylene C. S. Silva, Antonio Mendes-Sousa, Rafaela Paim, Gabriel C. A. Costa, Luciana R. Dias, Karla Oliveira, Mauricio R. V. Sant’Anna, Nelder F. Gontijo, Marcos H. Pereira, Grasielle D. Pessoa, Jesus G. Valenzuela, Leonardo B. Koerich, Fabiano Oliveira

**Affiliations:** 1 Laboratório de Fisiologia de Insetos Hematófagos, Departamento de Parasitologia, Instituto de Biologia, Universidade Federal de Minas Gerais, Belo Horizonte, Minas Gerais Brazil; 2 Departamento de Medicina, Universidade Federal do Piauí, Picos, Piaui Brazil; 3 Departamento de Bioquímica e Farmacologia, Centro de Ciências da Saúde, Universidade Federal do Piaui, Teresina, Piauí Brazil; 4 Section of Vector Biology, Laboratory of Malaria and Vector Research, National Institute of Allergy and Infectious Diseases, National Institutes of Health, Bethesda, Maryland United States of America

**Keywords:** Parasite genomics, RNA sequencing, Parasite biology

## Abstract

*Ornithodoros rostratus* is a South American argasid tick which importance relies on its itchy bite and potential as disease vector. They feed on a wide variety of hosts and secrete different molecules in their saliva and intestinal content that counteract host defences and help to accommodate and metabolize the relatively large quantity of blood upon feeding. The present work describes the transcriptome profile of salivary gland (SG) and midgut (MG) of *O. rostratus* using Illumina sequencing. A total of 8,031 contigs were assembled and assigned to different functional classes. Secreted proteins were the most abundant in the SG and accounted for ~67% of all expressed transcripts with contigs with identity to lipocalins and acid tail proteins being the most representative. On the other hand, immunity genes were upregulated in MG with a predominance of defensins and lysozymes. Only 10 transcripts in SG and 8 in MG represented ~30% of all RNA expressed in each tissue and one single contig (the acid tail protein ORN-9707) represented ~7% of all expressed contigs in SG. Results highlight the functional difference of each organ and identified the most expressed classes and contigs of *O. rostratus* SG and MG.

## Introduction

Argasid (soft) and ixodid (hard) ticks are hematophagous arthropods that feed on a wide variety of hosts. They are of medical and veterinary importance due to the blood spoliation and transmission of pathogens that cost significant financial losses worldwide in the animal industry^1,2^.

Unlike long term feeding hard ticks, in which feeding process can take up to two weeks, the soft ticks normally feed in minutes. In addition, while adults of hard ticks feed only once, adults of soft ticks are intermittent feeders, as they can take bloodmeal more than 10 times during their lifetime. As in all blood-sucking arthropods, to have a successful bloodmeal, ticks should bypass haemostatic, inflammatory and immune responses in the host’s skin^3^. This task is carried mainly by the salivary glands that produce and secrete several bioactive compounds into the host via saliva. The midgut also plays a key role in hematophagy. In addition to acting on the management of blood meal (storage and digestion) and protection against host immunity and pathogen infections, it has also been shown that the behaviour of blood in its lumen can affect the ingestion rate of the arthropod^4,5^. As some blood-sucking arthropods ingest with host blood part of the released saliva, it is likely that some of the biological activities present in the digestive system are due to salivary molecules. Digestion in ticks occurs intracellularly, inside lysosomal vesicles of midgut enterocytes, at acidic pH^6^. It goes through several modifications to accommodate and metabolize the relatively large amount of blood that it receives upon feeding. Although blood bolus volume is significantly reduced because of water excretion by the tick salivary (hard ticks) or coxal (soft ticks) glands, a tick will increase in total volume and mass by many times its original size and weight during a meal.

In hard ticks, which can remain attached for several days to the host to complete a blood meal, several thousands of unique contigs have being described in extensive sequencing transcriptomes^7^, which is believed to be the highest variety of proteins produced at the salivary glands among hematophagous arthropods. To study the variety of molecules related to the hematophagy, several transcriptomes of the salivary gland (SG) and midgut (MG) of hard ticks (at least 37 transcriptomes from 18 species) have been published^7,8^. On the other hand, the number of similar studies from ticks of the Argasid family is considerably lower. There are some data about the SG from *Ornithodoros moubata*, *O. parkeri*, *O. coriaceus*, *O. erraticus*, *Antricola delacruzi* and *Argas monolakensis* and few studies of MG transcriptomes that includes only *O. moubata*, *O. mimon* and *O. erraticus*^9–18^.

*Ornithodoros rostratus* is an argasid tick found in the South American countries Brazil, Paraguay, Argentina and Bolivia. They have a painful and itchy bite and are implicated as potential vectors of pathogenic bacteria as *Rickettsia rickettsii*, the causative agent of the South American Spotted Fever, and *Coxiella burnetii*, the etiological agent of Q Fever^19^. Even though, very little is known about the molecules present in their SG and MG of this species. The present work aimed at describing the transcriptome profile of SG and MG of *O. rostratus*. The diversity of molecules and the comparison of their level of expression in the two main organs involved in hematophagy are discussed throughout the study.

## Results and Discussion

### Transcriptome overview

Sequencing of SG and MG libraries produced 22,395,831 Illumina reads. De novo assembly generated 40058 putative contigs that were further selected down to 8031 contigs based on the presence of an ORF and any similarities to other proteins found in the Refseq invertebrate and Acari databases deposited at NCBI’s Genbank or at SwissProt database. The presence of a signal peptide was also considered as an additional positive selection criteria. Contig mean size in the final assembly is 1449.43 nucleotides long (largest contig is 29079 bases and the smallest is 155 bases). BUSCO analysis to access the transcriptome quality show complete BUSCOs of 85.6% (75.5% single-copy orthologs and 10.1% duplicated orthologs), 2.1% fragmented genes and 12.3% missing orthologs. Contigs were classified into six major categories namely housekeeping, secreted, immunity, transposable elements, viral and unknown (Supplementary Table 1). To visualize the differential expression of contigs, sequences were analysed using normalized transcripts per million (TPM). Such normalization reduced the biases caused by reads with few sequences and enables a more trustworthy comparison of contigs expression. The contigs classified as secreted were the most abundant with 886 contigs and 46.51% of the TPM, followed by housekeeping genes which had more contigs (5,325 contigs) but lower abundance (35.54%) of the TPM. Immunity related reads were assembled into 127 contigs and represented 8.41% of the TPM, while reads of the unknown category were assembled in 1,528 contigs but had lower levels of expression and their total TPM represented 9.29% of the library. Viral and transposable elements represented together 202 contigs (TPM of 2.51%) (Supplementary Table 1).

**Figure 1:**
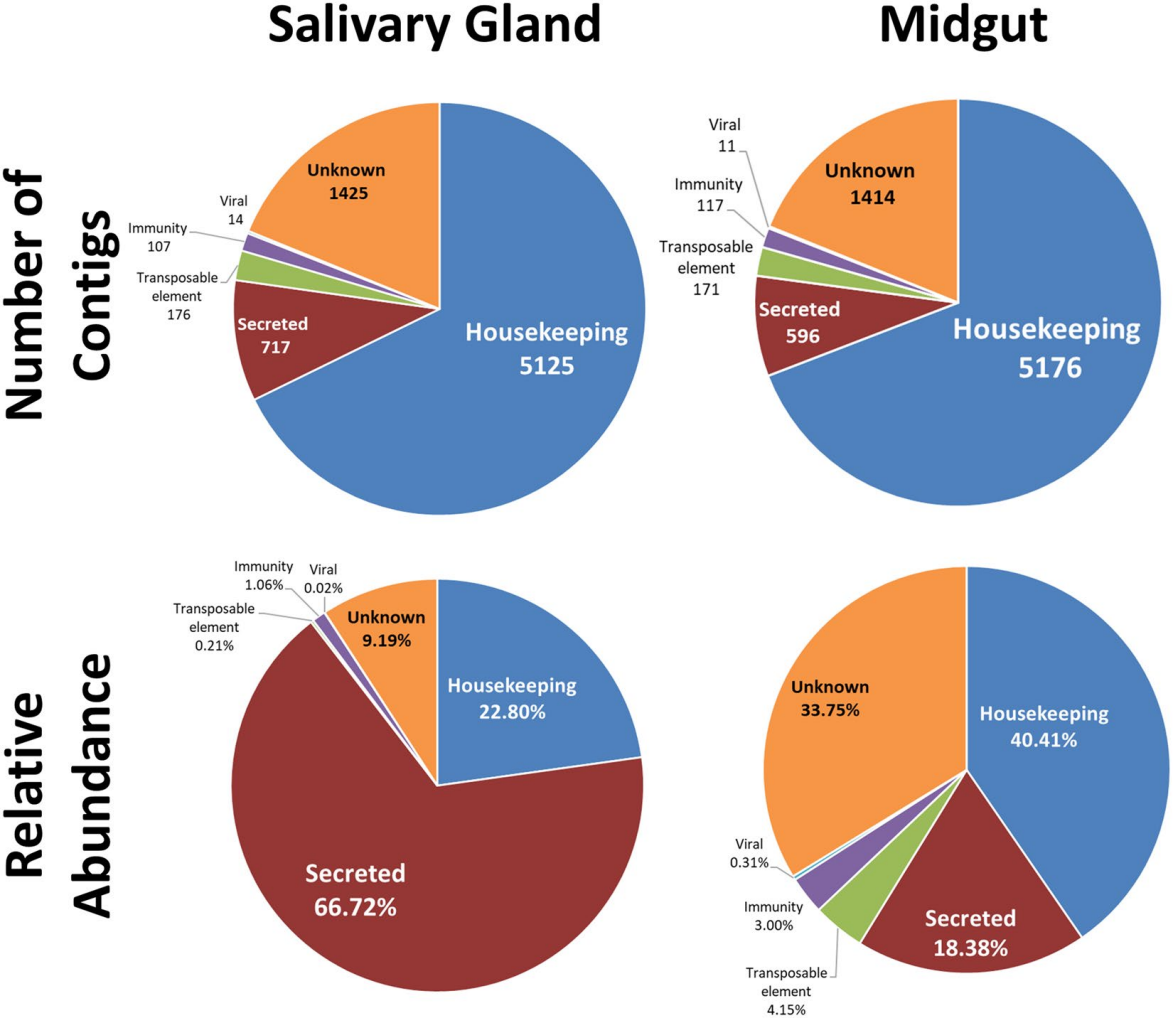
Dispersion of sequences based on number of contigs and TPM in salivary glands (SG) and midgut (MG) of *Ornithodoros rostratus*.

Housekeeping related transcripts accounted for almost 2/3 of the contigs, but the TPM corresponded to only 22.80% in the SG and 40.41% in the MG. Secreted transcripts were considerably more representative in the SG (717 contigs) but representing 66.72% of the TPM, while in the MG, the 596 contigs identified represented only 18.38%. On the other hand, immunity related transcripts had similar levels of contigs in both tissues but were ~2-fold more abundant in the MG in comparison to the SG (TPM of 3.00% and 1.06%, respectively). Other groups (viral, transposable element and unknown) were more abundant in MG than in SG, with an emphasis on transcripts with unknown function that had a high number of contigs (>1400) and were ~3-fold more representative in MG than in SG (Fig. 1).

### The topmost abundant transcripts in SG and MG

To find the main transcripts in each tissue, we ordered contigs by abundance (TPM) and evaluated those that, together, contributed to ~30% of all mRNA present in each tissue (Tables 1 and 2). In both cases, it is intriguing that so few transcripts (10 in SG and 8 in MG) could represent ~30% of all mRNA in each tissue. Nine out of the ten most expressed transcripts in SG belongs to the Secreted proteins group, distributed mostly in two functional classes - Acid Tail Proteins and Lipocalins (three transcripts each). The two most expressed SG transcripts were acid tail proteins (ORN-9707 and ORN-8689) representing more than 10% of all transcripts in SG (Table 1). Our data suggests that these transcripts are SG specific (we found no reads in the MG library – Supplementary dataset). The Lipocalins were also abundant with three contigs representing ~7% of the total SG TPM. The transcript ORN-13158 is the only secreted transcript with unknown function among the top ten expressed transcripts. However, BLAST and evolutionary analysis (Supplementary Fig. 1) strongly suggests that this transcript belongs to the 7DB-family of proteins and might be misannotated in the final transcriptome assembly.

**Table 1:** List of transcripts that represent ~30% of all RNA expressed in salivary glands.

Name	Abundance	TPM	Functional class/category	Accession
acid tail salivary protein	7.07%	71111.88	Acid tail proteins/Secreted	ORN-9707
acid tail salivary protein	3.76%	37806.78	Acid tail proteins/Secreted	ORN-8689
savignygrin	3.19%	32107.14	Desintegrin/Secreted	ORN-14583
moubatin	3.12%	31421.87	Lipocalins/Secreted	ORN-7555
acid tail salivary protein	2.85%	28628.95	Acid tail proteins/Secreted	ORN-15529
salivary secreted lipocalin	2.45%	24637.79	Lipocalins/Secreted	ORN-18741
salivary mucin	2.33%	23477.63	Mucin/Secreted	ORNSIGP-6290
Putative basic tail protein	2.22%	22332.1	Basic tail proteins/Secreted	ORN-4112
hypothetical protein	1.51%	15214.63	Unknown conserved/Unknown	ORN-13158
salivary lipocalin	1.47%	14808.19	Lipocalins/Secreted	ORN-4748

**Table 2:** List of transcripts that represent ~30% of all RNA expressed in midguts.

Name	Abundance	TPM	Functional class/Category	Accession
Defensin A	11.95%	123998.45	Defensin/Immunity	ORN-7204
Ferritin	5.13%	53206.36	Storage/Housekeeping	ORN-7176
Secreted protein	5.09%	52775.56	Hypothetical Conserved Secreted Proteins /Secreted	ORN-8595
Secreted protein PK-4 precursor	2.18%	22609.82	Hypothetical Conserved Secreted Proteins/Secreted	ORN-8761
Glutathione s-transferase D1	1.81%	18784.68	Detoxification/Housekeeping	ORN-18830
Cystatin precursor	1.64%	17019.2	Protease Inhibitors/Secreted	ORN-6013
Unknow salivary protein	1.63%	16909.78	Unknown product/Unknown	ORNSIGP-6180
Lysozyme precursor	1.48%	15398.43	Lysozyme/Immunity	ORNSIGP-6324

On the other hand, the MG had two immunity and two housekeeping transcripts among the most expressed, while the SG had none of those categories. Remarkably, a single defensin (ORN-7204) represents almost 12% of all transcripts in MG (total read count also suggests that this is the most abundant transcript in the whole dataset – see Supplementary dataset). Our data suggest that this defensin is not expressed in SG at all, playing a specific role in MG. It is also worth to mention the level of expression of a Ferritin (ORN-7176) in the MG, which is ~19x more abundant in MG than in SG.

### Functional coherence and abundance of transcripts by functional class in salivary gland and midgut

To globally visualize the families of contigs expressed in each tissue, we classified the transcripts of each major category into putative protein functional classes and evaluated the relative abundance of transcripts for each class in each tissue (Supplementary Tables 2–4). The Housekeeping contigs were attributed to 21 classes, while Secreted and Immunity contigs were attributed to 40 and 14 functional classes, respectively. Here, we briefly comment interesting findings in each major class.

#### Housekeeping

**Figure 2:**
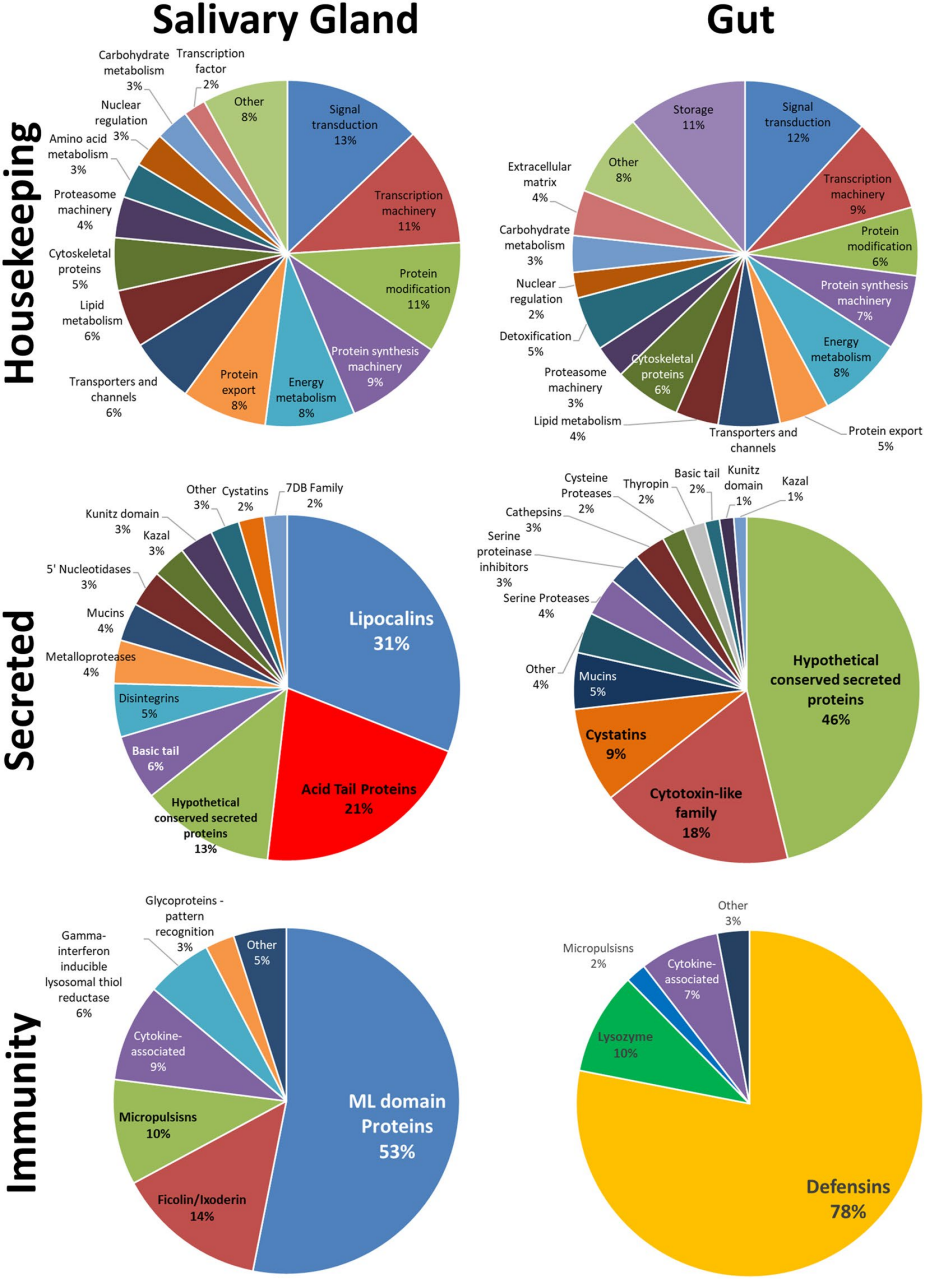
Relative abundance of transcripts by putative function for each major category in each tissue.

The TPM abundance for the housekeeping classes was relatively similar between tissues (Fig. 2 and Supplementary Table 2). The classes signal transduction and transcription Machinery were the ones with more contigs (1076 and 644, respectively) and the most abundant (~20% of all housekeeping TPM). The classes energy metabolism, protein synthesis machinery and protein modification were also among the six most abundant in both tissues. Storage transcripts were also abundant in the MG while protein export transcripts were abundant in the SG.

#### Secreted proteins

The expression profile in SG and MG for secreted genes was strikingly different (Fig. 2 and Supplementary Table 3). SG presented TPM almost 3-fold higher than the MG (692,248.44 × 258,101.68). It is remarkable that the number of hypothetical secreted proteins contigs is similar in both tissues (385 and 359 in SG and MG, respectively), but the overall abundance of such transcripts is considerably lower in the SG (~13%) in comparison to the MG (~46%). Acid tail proteins and lipocalins seems to have an important role in SG, since together these proteins represented more than 50% of the total SG TPM. On the other hand, the most abundant secreted protein class in MG was the cytotoxin-like family, which corresponded to ~25% of all the secreted TPM. A notable difference was also seen among the abundance of enzymes. SG had high levels of metalloproteases while cathepsins, cysteine proteases and serine proteases were more abundant in MG. Concerning the protease inhibitors, cystatins and proteins with Kunitz and Kazal domains were abundant in both tissues, while serine protease inhibitors and thyropin were also more represented in the MG (both with TPM above 1.5%).

#### Immunity

The number of immunity related contigs in each tissue was relatively similar, but the total TPM was ~15-fold higher in MG than in SG. Again, remarkable abundance differences were seen among the functional classes between tissues (Fig. 2 and Supplementary Table 4). ML-domain proteins in the SG and defensins in the MG are by far the most abundant immunity related transcripts in each tissue (53% and 78%, respectively). A single defensin transcript (ORN-7204) was responsible for ~45% of all immunity related transcripts in the MG. In addition to proteins with ML-domains, other four classes were well represented in the SG, as the ficolin/ixoderin, microplusins, gamma-interferon inducible lysosomal thiol reductase, cytokine associated and microplusins (which, together, accounts for >90% of immunity transcript abundance in SG). On the other hand, ~95% of the abundance of all immunity related transcripts in MG belonged to defensins, lysozymes and cytokine-associated proteins.

### Differential expression in salivary gland and midgut

**Figure 3:**
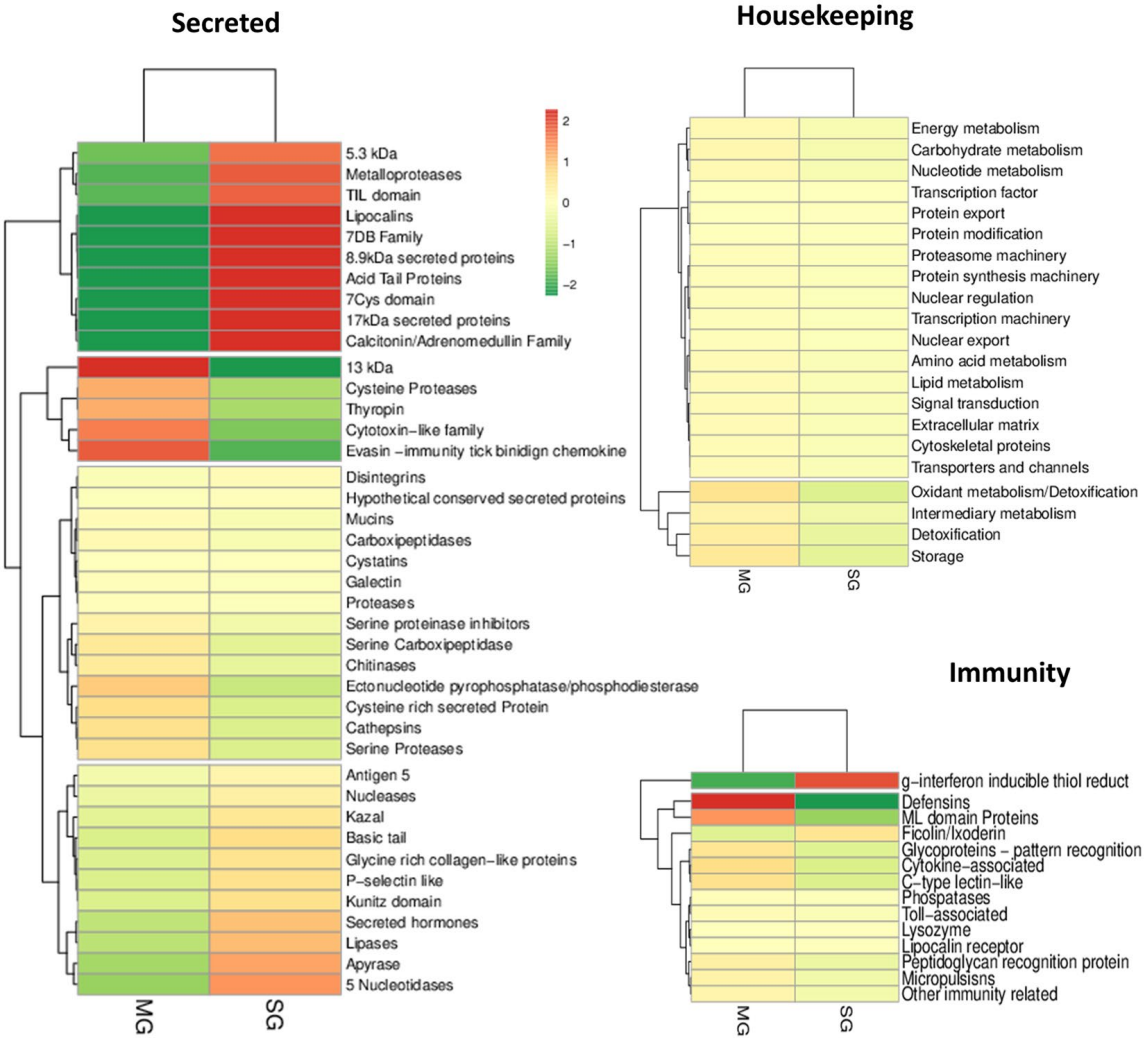
Heatmaps for each major functional category. Heatmaps show transcript abundancy (expression level) in each sample. In Bright Red are upregulated classes of genes (>2-fold standard deviation); Bright Green are downregulated classes of genes (<2-fold standard deviation); Brownish green or red are classes with standard transcription. Classes were grouped by a pattern of transcription (e.g. Genes that are upregulated in both tissues; genes that are differentially expressed, genes that are downregulated in both tissues; genes that has a standard regulation in both tissues; etc).

To have an insight in differential expression, the mean expression level (mean z-score) for each functional class in each tissue was evaluated. Heatmap analysis showed clear differences in the overall expression profile between SG and MG for secreted and immunity protein families, while there were no differences in the expression profile for housekeeping functional classes (Fig. 3). We found suggestive evidence of expression compartmentalization for some enzyme and protease inhibitor classes between organs. Metalloproteases and TIL-domain proteins were practically exclusive of the SG, while the cysteine proteases and thyropin were virtually only expressed in the MG.

Expression analysis of secreted proteins suggests that genes from eight other functional classes were upregulated (z-score > 2) in SG and downregulated in MG (z-score < −2), such as lipocalins, acid tail proteins, 7DB-family proteins, 7-cys domain proteins, calcitonin/adrenomedulin proteins, 17 kDa, 8.9 kDa and 5.3 kDa secreted proteins. On the other hand, 13 kDa secreted proteins, cytotoxin- like and evasin have expression clearly upregulated in MG and downregulated in SG.

For immunity related transcripts we observed expression differences in defensins and ML-domain proteins which were considerably more expressed in MG than in SG, while G-interferon inducible thiol reductase seemed to be differentially more expressed in SG. It is important to note the case of ML-domain proteins. Heatmap analysis suggests that transcripts of this family are, on average, more expressed in MG than in SG (z-scores of 0.74 and −1.10, respectively). On the other hand, transcript abundance based on TPM shows that ML-domain transcripts represent ~53% of all immunity related transcripts in SG, while it accounts for less than 1% of the immunity transcripts in MG. We found a total of six ML-domain transcripts in the *O. rostratus* transcriptome. All six are expressed in the MG (TPM values from 4.62 to 528.37 with three of them with TPM above 200). On the other hand, only three ML-domain transcripts are expressed in SG (two of them with TPM bellow 40) and the ORN-33642 has a TPM of 5768.55. This single ML-domain transcript account for ~53% of all immunity related genes transcribed in the SG.

### Discussion on the main classes of secreted protein

#### Lipocalins

Proteins of the lipocalin family are known mainly for their role as carriers and ligands of soluble molecules, especially hydrophobic molecules^20^. They are expressed in the SG of insects and ticks and can perform a variety of functions. Lipocalins are particularly abundant in triatomine transcriptomes where they usually represent more than 50% of the secreted proteins, reaching up to 90% in some species^21^. In ticks, lipocalins are produced in the SG of all studied species, with described functions that varies from interfering with host hemostasis^22,23^, inhibition of complement factor C5 activation^24^, platelet and neutrophil aggregation inhibition^25,26^ and inhibition of histamine-mediated inflammation^27^. The family diversification in ticks has been observed, with the number of lipocalins genes ranging between 34 to 52^25,28^.

**Figure 4:**
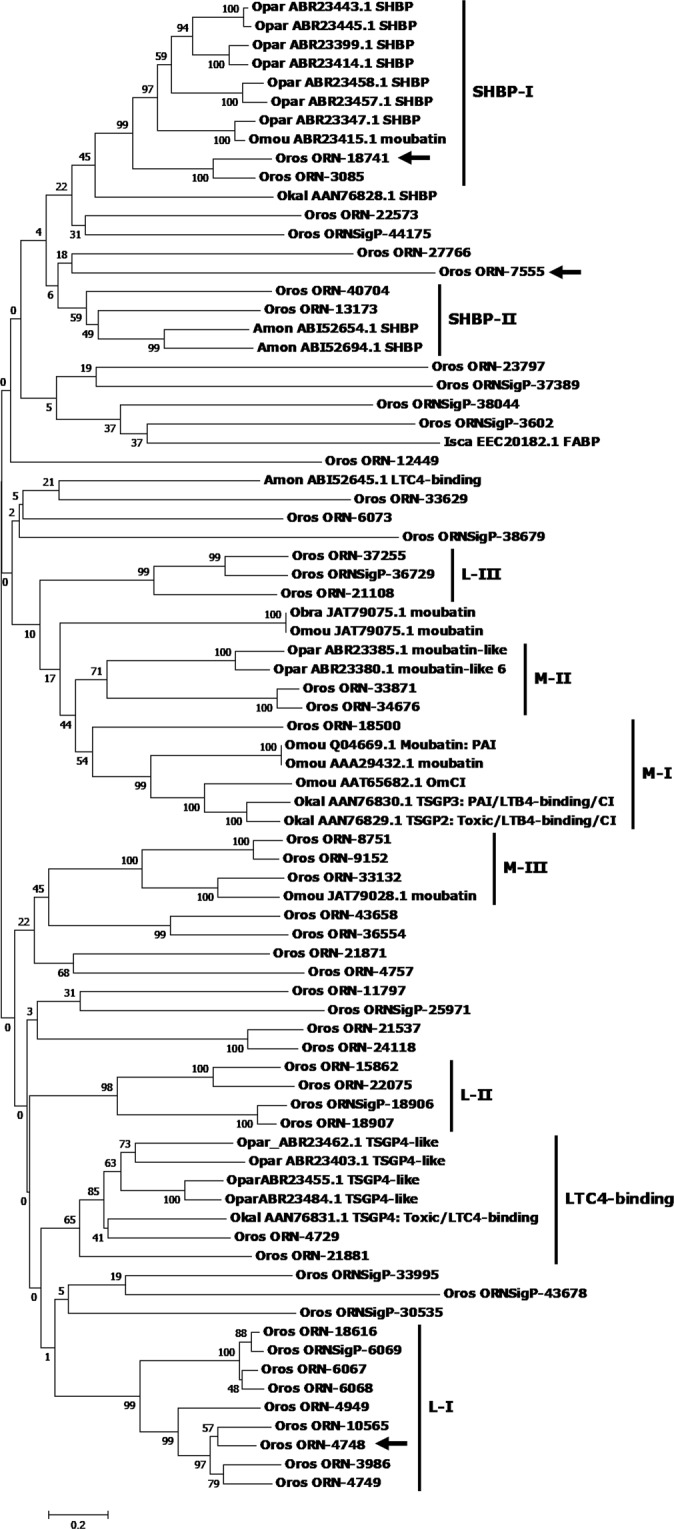
Evolutionary relationships of *Ornithodoros rostratus* Lipocalins. The evolutionary history of the *O. rostratus* lipocalins was inferred using the Neighbour-Joining method. The optimal tree with the sum of branch length = 40.29 is shown. The analysis involved 72 amino acid sequences. There was a total of 386 positions in the final dataset. Evolutionary analyses were conducted in MEGA. Oros = *Ornithodoros rostratus*; Opar = *O. parkeri*; Ocor = *O. coriaceus*; Omou = *O. moubata*; Okal = *O. kalahariensis*; Amon* = Argas monolakensis*; Isca = *Ixodes scapularis*. L-I to L-III: lipocalin clades I to III; M-I to M-III: moubatin clades I to III; SHBP-I and II = serotonin and histamine biding proteins clades I and II; LTC4 = leukotriene-C_4_ (cysteinyl rich leukotrienes); PAI = platelet aggregation inhibitor; CI = complement inhibitor; LTB4 = leukotriene-B_4_. Black arrows indicate the three most expressed lipocalins in the salivary gland.

Almost 31% of all SG secreted proteins corresponded to lipocalins, which demonstrates the importance of these molecules. Of the 48 lipocalins found in *O. rostratus*, 25 of them seem to be differentially expressed in the SG (z-scores > 1.8; Supplementary dataset), while 32 of them were not expressed in MG at all (TPM = 0.0). Phylogenetic analysis suggests at least four expansions of this family in *O. rostratus* (Fig. 4, clades LI to LIII and MIII). Our phylogenetic analysis suggests at least six *O. rostratus* lipocalins belongs to the moubatin family (Fig. 4), and these proteins could be classified in three distinct groups (MI to MIII). One sequence in specific (ORN-18500) is more related to moubatins from the MI clade. Functional studies showed that moubatins from this clade have roles in the inhibition of the activation of C5 of vertebrate complement system^24^; platelet aggregation inhibition by scavenging thromboxane A_2_ and neutrophil aggregation inhibition by scavenging leukotriene B_4_^25,29^. Other two contigs (ORN-4729 and ORN-21881) seems to be related to TSGP-4 family (tick salivary gland peptide 4) which are implicated in the scavenging of cysteinyl rich leukotrienes^26^. Cysteinyl rich leukotrienes C4, D4 and E4 (LTC4, LTD4 and LTE4) are produced by mast cells and basophils and have a role as mediators of inflammation. We also found four *O. rostratus* lipocalins distributed into two clades of serotonin and histamine biding proteins (ORN-18741 and ORN-3085 in the clade SHBP-I and; ORN-40704 and ORN-13173 in the clade SHBP-II). SHBPs were already described in the saliva of soft and hard ticks and their ability to scavenge serotonin and histamine indicates they suppress inflammation during blood feeding^27,30–32^. Interestingly, the lipocalin ORN-18741 (related to SHBP-I) is among the top most expressed genes in *O. rostratus* salivary glands (Table 1). On the other hand, the other two highly expressed lipocalins (ORN-7555 and ORN-4748) does not seems to belong to any lipocalin family with known function.

#### Acid tail transcripts

**Figure 5:**
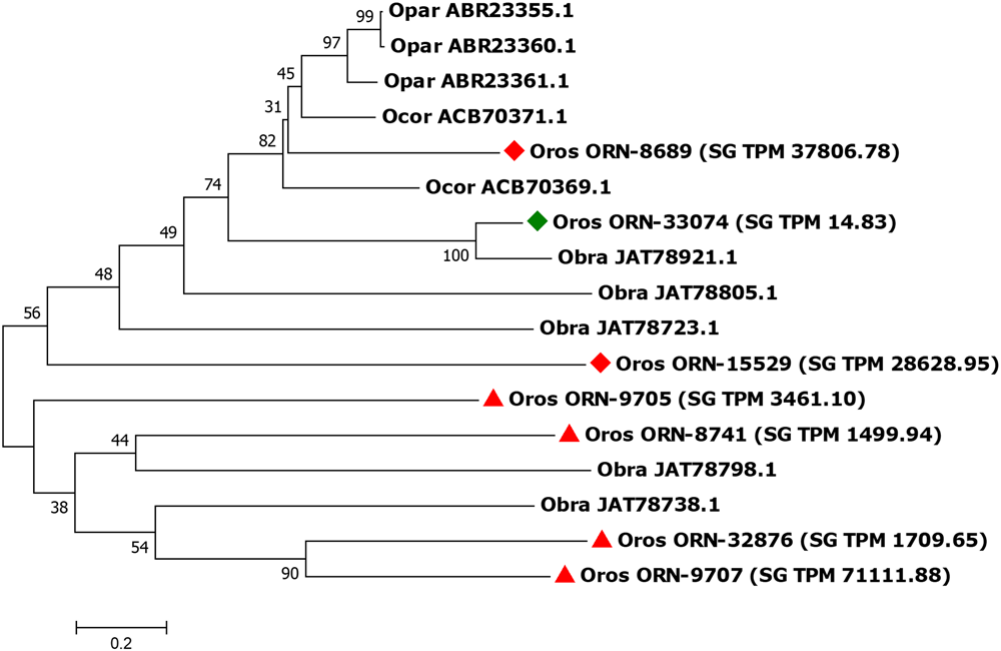
Evolutionary relationships of *Ornithodoros rostratus* acid tail proteins. The evolutionary history of the *O. rostratus* acid tail proteins were inferred using the Neighbour-Joining method. The optimal tree with the sum of branch length = 11.45 is shown. The percentage of replicate trees in which the associated taxa clustered together in the bootstrap test (10000 replicates) are shown next to the branches. The analysis involved 17 amino acid sequences. All ambiguous positions were removed for each sequence pair. There was a total of 334 positions in the final dataset. Red triangles indicate specific salivary gland (SG) overexpressed transcripts, red diamonds indicate transcripts overexpressed in SG in comparison to midgut (MG) and green diamonds indicate low expression in both tissues. Oros = *Ornithodoros rostratus*; Opar = *O. parkeri*; Obra = *O. brasiliensis*, Ocor = *O. coriaceus*. SG transcripts per million (TPM) is indicated within round brackets.

Acid tail proteins are molecules with unknown function that shares a common characteristic of having a predominance of glutamate. Acid tail proteins were overexpressed only in SG. Overexpression of acid tail proteins was also reported in *I. ricinus*, with expression levels > 10-fold higher in SG compared to the MG^33^. From the seven acid tail transcripts of *O. rostratus*, four showed differential expression in SG with z-scores > 2.0 (Supplementary dataset). All the seven acid tail proteins presented a conserved PFAM domain named TSGP1 (tick salivary peptide group 1). One acid-tail protein, ORN-9707, caught our attention as the most abundant transcript in SG. This transcript showed a z-score of 3.09, corresponding for > 7% of all SG transcripts, and for ~10% of all secreted transcripts. Phylogenetic analysis suggests that ORN-9707 is a duplication of another acid tail protein (ORN-32876) and a possible ortholog of *O. brasiliensis* acid tail proteins JAT78798 and JAT78738 (Fig. 5). The acid tail protein ORN-32876 showed a z-score = 1.80. Such relationship could indicate a recent duplication with possible selection for the ORN-9707. However, our data do not allow us to speculate further.

#### Basic Tail Proteins

Basic tail proteins represented 6.11% and 1.38% of the secreted transcripts in the SG and MG, respectively (Supplementary Table 2). The name “basic tail” is related to a cluster of basic amino acids at the carboxy terminal end of the protein. This family was already reported for both soft and hard ticks^34^. In argasids, this class was also abundant in the saliva of *O. coriaceus* and *O. parkeri*^11,12^. From the nine *O. rostratus* basic tail contigs, ORN-24121 and ORNSIGP-21115 seems to be differentially expressed in MG (z-scores > 1.8) and presented similarities with RNA recognition motif-containing protein and secreted PAPA repeat protein, respectively. In SG, three basic tail transcripts showed z-scores > 2.0 (ORN-21032; ORN-4112 and ORN-10496).

#### Mucins

Similarly, to basic tail proteins, mucins were overexpressed in both organs. We found 28 contigs and two of them presented high level of expression in SG (ORNSIGP-6290) or MG (ORNSIGP-25718). Mucins are proteins with a C-terminal serine-threonine rich domain capable of binding to N-acetyl-galactosamine^35^. After glycosylation, mucins are incorporated to a mucus produced by the saliva of ticks that has a possible function as a coating layer and, consequently, protection of buccal parts during blood feeding, besides the possible interaction with proteins of the host extracellular matrix^34^. The presence of salivary mucins has been observed in sialomes of other hematophagous arthropods, among them species of ticks of the genus *Ornithodoros* as *O. parkeri* and *O. coriaceus*^11,12^.

#### Enzymes

High number of enzyme transcripts is a common finding in the SG and MG transcriptomes of all studied ticks^11,12,33,36–38^. In *O. rostratus*, enzymes were highly represented in both tissues and a notorious distinction of classes were seen between SG and MG. Metalloproteases was the most expressed protein subclass in the SG (Fig. 3) while cysteine proteases, cathepsin and serine proteases were more expressed in the MG. Metalloproteases are proteolytic enzymes that require a metal ion (Zn^2+^) for catalysis reactions and are very abundant in the venom of animals^39^. The main purpose of metalloproteases in the tick SG would be the degradation of the host tissues at the bite site to form the feeding pool. This class of enzyme is also active in the degradation of fibrinogen and fibrin thus avoiding blood coagulation and maintaining the fluidity of blood^40^. They have a key role in tick feeding and have been described as a potential antigen to be used as a tick vaccine^41^.

On the other hand, the main purpose of the enzymes produced in the MG is the digestion of the diet. Digestion in ticks occurs intracellularly in lysosomes^6^ by a multi enzymatic cascade^42^. Several midgut proteases of ixodids^43^ and argasids of the genus *Ornithodoros*^44,45^ have already being identified. Three enzyme classes were highly expressed in the MG: cathepsin, serine proteases and cysteine proteases. Those protease classes were also upregulated in the midgut of *O. erraticus* 24 hrs after blood ingestion^16^. Among the *O. rostratus* cathepsins, types B, D, I and F were described, and the most abundant ones were two transcripts with 71% identity to *I. scapularis* Cathepsin B endopeptidase and with 57% identity to *I. ricinus* cathepsin D.

#### Protease inhibitors

Secreted protease inhibitors were also well represented in the *O. rostratus* SG and MG. Their high expression levels reflect the importance of this class involved in blocking (or reducing) host reparatory responses, signal pathways or activation cascades triggered by the feeding process. We found 24 protease inhibitors transcripts containing kunitz domains (five of them with two kunitz domains). Protease inhibitors with a kunitz domain are usually responsible to block serine proteases involved in host hemostasis^46^. Kunitz-type domain is characterized by short length (usually 50–60 amino acids), low molecular weight (6 kDa) and six invariantly spaced cysteine residues^47^. Sequences for proteins containing kunitz domains are frequently and abundantly found in tick sialotranscriptomes^11,34,36^. Saliva of *O. moubata* and *O. kalahariensis* contain, respectively. ornithodorin and savignin proteins presenting two kunitz domains that are inhibitors of thrombin activity^48,49^. In the hard tick *I. scapularis* there is two proteins, ixolaris and penthalaris, with kunitz domains that inhibit blood clotting by binding to factor Xa^50,51^. Other three protease inhibitor proteins were characterized as papilins. Papilins have been implicated in interacting with metalloproteases in *Drosophila melanogaster* and *Caenorhabditis elegans* as essential for embryonic development^52^. The presence of papilins in SG suggests a possible new role for these proteins.

Most of the other protease inhibitor contigs belonged to the classes of the serine protease inhibitors (Serpins), cystatins and thyropin and were all highly expressed in the MG. Those were also the main classes of protease inhibitors found in *O. mimon* MG^15^. Serpin domains were also found in MG of *O. moubata*^9^. Serpins have important roles in the modulation of tick-host interactions, such as suppression of host’s immune system and blood coagulation^53,54^. In the MG, those activities should be suppressed in the ingested blood in order to accommodate the blood ingested upon feeding^4^ and avoid immune responses against the gut wall^55^. Cystatins are involved in different biological processes, such as modulation of endogenous proteolysis, immune response and blood feeding^56^. In *O. moubata*, the cystatins have been described in the salivary glands and midgut, presenting important role in evasion of the host’s immune system and midgut physiology. A salivary cystatin from *O. moubata* (OmC2) is responsible for suppressing host’s immune system, inhibiting cathepsins and suppressing antigen presentation by dendritic cells, reducing the production of proinflammatory cytokines and T CD4 + cell proliferation^57^. Midgut cystatins of *O moubata* are inhibitors of cathepsins B and H, involved in the regulation of proteolytic targets in the tick digestive system and modulation of host immune response during blood feeding^58^. One contig coding a protein from the thryropin class was highly expressed in *O. rostratus* MG (ORN-37269; z-score of 2.18) (Supplementary table Excel). Thyropin is presented as a repeat of the amino terminal region of human thyroglobulin. These domains are normally described as cysteine-protease inhibitors and binding partners of heparin^59^. They have been found in transcriptomes of ticks’ SG and MG, including the *Ornithodoros* genus^15,60^. Their function for ticks, however, remains unknown.

### Discussion on the main classes of immunity transcripts

Six classes from the immunity related contigs were considered more representative in the SG or MG, which contained transcripts mostly involved in pathogen/antigen recognition, signalling of immune pathways or direct antimicrobial activity. Microplusins and cytokine-Associated were the classes with abundant contigs in both tissues, while ML-domain proteins and ficolins/ixodegrins were more abundant in the SG and defensins and lysozymes were more abundant in the MG (Fig. 2 and Supplementary Table 4). Interestingly, we observed that only three and four contigs accounted for ~75% (SG) and ~95% (MG) of the overall abundance of immunity transcripts in each tissue.

In the SG, the most abundant immunity related transcript was a ML-domain protein with homology (>70% amino acid identity) to a salivary lipid interacting protein from *O. parkeri* and *O coriaceus*. ML-domain proteins interact with specific lipids and have roles in the recognition of pathogens^61^. The other two abundant contigs in *O. rostratus* SG were a lectin of the ficolin class with 67% identity to *O. moubata* dorin M precursor and a microplusin highly expressed in both tissues (z-scores of 1.65 and 2.01 in SG and MG, respectively). The microplusin (ORN-44579) was homologous (>70% amino acid identity) to a hebrain-like protein from *O. coriaceus* and *O. parkeri*. Hebrain, from the ixodid tick *A. hebraeum*^62^, and microplusin, from *R. microplus*^63^, are antimicrobial peptides. Microplusin was shown to be a copper chelating peptide active against fungi and bacteria^64^.

Among the four contigs highly expressed in the MG, one (ORN-7204) showed ~80% amino acid identity with *O. moubata* defensin A. This transcript represented more than 75% of the total abundance of immunity related transcripts in MG and is the most abundant transcript in the MG transcriptome (12% of MG TPM). Defensins are one of the most important and widespread antibacterial peptides from invertebrates^65^. The mature defensins from *O. moubata* contain six conserved cysteine residues and have high identity to other scorpion defensins, but at a lesser degree to defensins of dragonflies and other ancient arthropods. In *O. moubata*, the defensin mRNA expression was up-regulated by blood-feeding^66^.

The second most abundant immunity related transcript in MG is a lysozyme precursor (ORNSIGP-6324). Together with the defensin, those two contigs accounted for almost 90% of the MG Immunity-related TPM. Lysozymes are bacteriolytic peptides which are part of the innate immunity of several groups of animals and plants^67^. The structure of this lysozyme indicates it is a typical Tick Gut Lysozyme (TGL), which are peptides also involved in digestion and with structure characteristic of c-type lysozymes that contain eight cysteine residues^68^. TGLs are also characterized by the presence of a histidine at position 52 which replaces the highly conserved tyrosine found in most c-type lysozymes. As defensins, the expression of TGL in the MG is also stimulated by the ingestion of blood in *O. moubata*^69^.

The other two of the most expressed immunity transcripts were classified as putative tumour necrosis factor receptor attributed to the Cytokine-associated class. TNF and their receptors are involved in immunity processes once they participate in several cellular signalling pathways that induce cell proliferation, survival, and differentiation processes^70^.

### Final insights in the *O. rostratus* transcriptome

The analysis of the salivary gland and midgut transcriptome of *O. rostratus* can give us some insights on: (*i)* the role of each organ in the tick physiology; (*ii)* similarities and differences of both organs on gene expression; (*iii)* key-role genes for a successful bloodmeal; (*iv)* new targets for further functional studies and; (*v)* possible targets for tick vaccines. The first and most clear observation is the functional coherence of each organ. Our data shows that SG excels in the production of secreted proteins, especially those correlated to maintain homeostasis and recognition of antigens during blood-feeding (e.g. lipocalins, metalloproteases, kunitz-domain proteins, apyrases). On the other hand, besides blood digestion, the MG intensively produces immunity related genes (e.g. Defensins, lysozymes) suggesting the importance to control bacteria growth during blood digestion. A few genes caught our attention due to their high abundance in each tissue. Lipocalins and acid-tail proteins are the most predominant transcripts in the SG. While the role of lipocalins to maintain homeostasis during blood ingestion is well known, the role of acid-tail proteins in this process (and one acid-tail protein is the most abundant transcript in SG) need further studies. In MG, a single defensin-A is the most abundant gene in all transcriptome and, although the role of defensins is well known in other arthropods, new studies on the role of this gene in *O. rostratus* are needed. Such observations indicate those genes are extremely important for tick feeding and may constitute important targets to be focused on control programs such as vaccine antigens or drug targets.

The current study brings new knowledge on salivary and intestinal molecules of argasid ticks, specially from *O. rostratus*, which gene information in databases are scarce. Although this study has its limitations (e.g. expression is based in a pool of individuals in different physiological stages and lack of samples for statistical analysis on differential expression), the intention was to show an overview of molecules produced in the SG and MG of *O. rostratus*. The information provided by this study will help in the design of future experiments to better understand the role of the highly expressed genes and to improve our knowledge on the biology *O. rostratus* and soft ticks.

## Methods

### Ticks

Ticks were obtained from a colony maintained at the Department of Parasitology – UFMG. The colony was established in 2010 from specimens collected at Nhecolândia, Mato Grosso do Sul, Brazil (19″03′S, 56″47′W). Ticks are reared inside an incubator under semi controlled conditions of temperature (28 ± 2 °C) and humidity (85 ± 10%) and fed on Swiss mice (*Mus musculus*) every 20 days^71^. Mice were maintained according to the regulations of the ethical committee in animal experimentation of UFMG (Comite de Etica no Uso de Animais – CEUA) and all experiments were approved by the same committee (protocol number 301/2013).

### Salivary gland and midgut isolation

SG and MG were dissected from unfed and one and three-day fed fourth instar nymphs (two of each). Tick lateral cuticule was cut with micro scissors, the superior cuticule was folded up front and the interior organs of ticks were exposed. Tissues were collected with forceps, rinsed by immersion in saline solution (NaCl 0.9%), transferred to tubes containing 50 µL of RNA latter (Sigma Aldrich) and stored at 4 °C until use.

### Sequencing

Samples were homogenized by multiple passages through a sterile 18G needle attached to a 1 mL syringe. Messenger RNA was isolated from SG and MG from *O. rostratus* using FastTrack® MAG mRNA Isolation Kits (ThremoFisher) and measured in a Bioanalyzer 2100 (Agilent Genomics). Samples were sent to the North Carolina State Genomic Sciences Laboratory (Raleigh, NC, USA) for Illumina RNA library construction and ran on an Illumina HiSeq 2500 DNA sequencer, using 125 bp single end sequencing flow cells following the manufacturer directions.

### Bioinformatic tools and procedures

Custom bioinformatic analysis were describe elsewhere^72^ with modifications. Concisely, low quality reads were trimmed from Fastq files (<20) and contaminating adapter primer sequences removed. De novo assembly from reads was a result of Abyss (using k parameters from 21 to 91 in 5-fold increments)^73^ and SOAP de novo-trans^74^ assemblers. The combined fasta files were further assembled using an iterative blast and CAP3 pipeline as previously described^75^. The final transcriptome quality was quantitatively assessed using Benchmarking Universal Single-Copy Orthologues (BUSCO) v 3.0^76^ against the arthropod database v9.0 with default parameters. Coding sequences (CDs) were extracted based on the existence of a signal peptide in the longer open reading frame (ORF) and by similarities to other proteins found in the Refseq invertebrate database from the National Center for Biotechnology Information (NCBI), proteins from Acari deposited at NCBI’s Genbank and from SwissProt. Only contigs containing open reading frame or any similarity to sequences in the chosen databases were selected for further analysis (please see references^72,75^ for details). Reads for each library were mapped on the deducted CDs using blastn with a word size of 25, 1 gap allowed and 95% identity or better required. Up to five matches were allowed if and only if the scores were the same as the largest score. Mapping of the reads was also included in the Excel spreadsheet. Values of the reads per kilobase of transcript per million mapped reads (TPM)^77^ for each coding sequence were also mapped to the spreadsheet. Automated annotation of proteins was based on a vocabulary of nearly 350 words found in matches to various databases, including Swissprot, Gene Ontology, KOG, Pfam, and SMART, Refseq-invertebrates and the Acari subset of the GenBank sequences obtained by querying acari [organism] and retrieving all protein sequences. Raw reads were deposited on the Sequence Read Archive (SRA) of the National Center for Biotechnology Information (NCBI) under bioproject ID PRJNA270484. This Transcriptome Shotgun Assembly project has been deposited at DDBJ/EMBL/GenBank under the accession GCJJ00000000.1.

### Differential expression analysis

For differential expression analysis, TPM values were transformed into z-scores, using data from both tissues to calculate the mean Log. For heatmap analysis, we calculated the mean z-score for each protein class based on their putative role (e.g. protein synthesis machinery, amino-acid metabolism, detoxification, etc). Heatmap of protein classes was constructed using Heatmapper^78^. Since we were interested only in having an insight in contig abundancy, we did not perform in depth differential expression analysis using common approaches (e.g. DEGseq or edgeR). Thus, we do not present and discuss any DE contigs in terms of statistical significance.

### Evolutionary analysis

Protein sequences from other organisms were obtained at NCBI and aligned with Muscle^79^. Evolutionary analyses were conducted in MEGA7^80^. The evolutionary history was inferred using the Neighbour-Joining method (10.000 replicates; pairwise deletion)^81^. The evolutionary distances were computed using the Poisson correction method and are in the units of the number of amino acid substitutions per site. All accession numbers are shown in the respective figures.

## Supplementary information

Supplementary information

Dataset

## Data Availability

All sequences and annotation tables are freely available in the GenBank or as the Supplementary dataset.
