# Supplementary material for: RNA-seq analysis of the salivary glands and midgut of the Argasid tick Ornithodoros rostratus
Source: Sci Rep. 2019 May 1;9:6764. doi: 10.1038/s41598-019-42899-z (PMC6494864; doi:10.1038/s41598-019-42899-z)
Supplement: Supplementary file 1 — Supplementary information [file 41598_2019_42899_MOESM1_ESM.docx]

RNA-seq analysis of the salivary glands and midgut of the Argasid tick *Ornithodoros rostratus*

Ricardo N Araujo^1+^; Naylene C S Carvalho^1^; Antonio Mendes-Sousa^1,2^; Rafaela Paim^1^; Gabriel C A Costa^1^; Luciana R Dias^1^; Karla Oliveira^3^; Mauricio RV Sant’Anna^1+^; Nelder F Gontijo^1+^; Marcos H Pereira^1+^; Grasielle D Pessoa^1^; Jesus G Valenzuela^4^; Leonardo B Koerich^1+*^; Fabiano Oliveira^4*^

^1^Laboratório de Fisiologia de Insetos Hematófagos, Departamento de Parasitologia, Instituto de Biologia, Universidade Federal de Minas Gerais, Belo Horizonte, Minas Gerais, Brazil.

^2^Departamento de Medicina, Universidade Federal do Piauí, Picos, Piaui, Brazil.

^3^Departamento de Bioquímica e Farmacologia, Centro de Ciências da Saúde, Universidade Federal do Piaui, Teresina, Piauí, Brazil.

^4^Section of Vector Biology, Laboratory of Malaria and Vector Research, National Institute of Allergy and Infectious Diseases, National Institutes of Health, Bethesda, Maryland, United States of America.

^+^Instituto Nacional de Ciência e Tecnologia – Entomologia Molecular.

*Corresponding authors: L.F.O. (email: loliveira@niaid.nih.gov) and L.B.K. (email: [lbkoerich@ufmg.br](mailto:lbkoerich@ufmg.br)).

# **Supplementary Material**

**Supplementary Table 1.** Major categories of CDS extracted from salivary glands and midgut of *Ornithodoros rostratus*.

| **Category** | **Contigs** | **% Contigs** | **TPM** | **%TPM** |
| --- | --- | --- | --- | --- |
| Housekeeping | 5325 | 66.31% | 726187.24 | 35.54% |
| Secreted | 886 | 11.03% | 950350.12 | 46.51% |
| Immunity | 127 | 1.58% | 171811.21 | 8.41% |
| Unknown | 1528 | 19.03% | 189906.9 | 9.29% |
| Viral | 14 | 0.17% | 495.52 | 0.02% |
| Transposable element | 188 | 2.34% | 4627.72 | 0.23% |
| **Total** | **8,031** | **100.0** | **2043378.71** | **100.00%** |

| **Supplementary Table 2. Housekeeping proteins** | | | |  |  |  |  |
| --- | --- | --- | --- | --- | --- | --- | --- |
| **Functional Family** | **Contigs** | **Contigs SG** | **TPM SG** | **%SG** | **Contigs MG** | **TPM MG** | **% MG** |
| Signal transduction | 1076 | 1026 | 30520.44 | 12.90% | 1040 | 57817.17 | 11.81% |
| Transcription machinery | 644 | 637 | 26235.10 | 11.09% | 641 | 43339.11 | 8.85% |
| Transporters and channels | 380 | 354 | 14476.24 | 6.12% | 355 | 28170.21 | 5.75% |
| Protein export | 362 | 361 | 18814.09 | 7.95% | 357 | 22771.13 | 4.65% |
| Protein modification | 324 | 311 | 24602.20 | 10.40% | 309 | 31248.13 | 6.38% |
| Nuclear regulation | 292 | 292 | 7642.52 | 3.23% | 289 | 11754.09 | 2.40% |
| Cytoskeletal proteins | 267 | 261 | 11677.11 | 4.94% | 262 | 30490.98 | 6.23% |
| Lipid metabolism | 259 | 244 | 12816.17 | 5.42% | 244 | 19627.88 | 4.01% |
| Proteasome machinery | 257 | 256 | 9072.23 | 3.83% | 256 | 15087.81 | 3.08% |
| Carbohydrate metabolism | 208 | 196 | 7371.36 | 3.12% | 202 | 16727.09 | 3.42% |
| Energy metabolism | 192 | 188 | 19789.56 | 8.36% | 191 | 39302.49 | 8.03% |
| Transcription fator | 184 | 178 | 4895.26 | 2.07% | 171 | 10442.54 | 2.13% |
| Protein synthesis machinery | 183 | 183 | 22021.66 | 9.31% | 182 | 34390.99 | 7.02% |
| Extracellular matrix | 171 | 160 | 4753.19 | 2.01% | 163 | 20881.81 | 4.27% |
| Nucleotide metabolism | 141 | 133 | 2558.05 | 1.08% | 139 | 4966.45 | 1.01% |
| Amino acid metabolism | 113 | 105 | 7813.13 | 3.30% | 107 | 10605.98 | 2.17% |
| Detoxification | 100 | 86 | 4010.88 | 1.70% | 99 | 24769.81 | 5.06% |
| Oxidant metabolism/Detoxification | 62 | 49 | 2255.46 | 0.95% | 59 | 8770.77 | 1.79% |
| Intermediary metabolism | 57 | 52 | 808.79 | 0.34% | 57 | 2643.07 | 0.54% |
| Nuclear export | 34 | 34 | 594.63 | 0.25% | 34 | 980.80 | 0.20% |
| Storage | 19 | 19 | 3861.18 | 1.63% | 19 | 54809.68 | 11.19% |
| **Total Housekeeping** | **5325** | **5176** | **236589.25** | **100.00%** | **5125** | **489597.99** | **100.00%** |
|  |  |  |  |  |  |  |  |

| **Supplementary table 3. Secreted proteins** | | | | | | | |
| --- | --- | --- | --- | --- | --- | --- | --- |
| **Functional family** | **Contigs** | **Contigs SG** | **TPM SG** | **%SG** | **Contigs MG** | **TPM MG** | **% MG** |
| Lipocalins | 48 | 48 | 214528.28 | 30.99% | 16 | 305.94 | 0.12% |
| Mucins | 28 | 23 | 24782.68 | 3.58% | 23 | 13584.33 | 5.26% |
| Cytotoxin-like family | 18 | 8 | 294.95 | 0.04% | 18 | 46901.56 | 18.17% |
| 7DB Family | 13 | 13 | 15126.57 | 2.19% | 1 | 18.71 | 0.01% |
| Basic tail | 9 | 6 | 42273.52 | 6.11% | 4 | 3551.41 | 1.38% |
| Disintegrins | 9 | 6 | 34553.05 | 4.99% | 6 | 1189.69 | 0.46% |
| 5' Nucleotidases | 7 | 7 | 23775.73 | 3.43% | 5 | 132.47 | 0.05% |
| Acid Tail Proteins | 7 | 7 | 144233.13 | 20.84% | 3 | 10.65 | 0.00% |
| Antigen 5 | 6 | 6 | 1896.85 | 0.27% | 5 | 969.11 | 0.38% |
| Glycine rich collagen-like proteins | 6 | 6 | 1403.88 | 0.20% | 5 | 1167.84 | 0.45% |
| Calcitonin/Adrenomedullin Family | 6 | 6 | 3210.39 | 0.46% | 0 | 0.00 | 0.00% |
| Secreted hormones | 6 | 5 | 129.06 | 0.02% | 2 | 30.75 | 0.01% |
| Apyrase | 3 | 3 | 605.45 | 0.09% | 1 | 24.31 | 0.01% |
| Galectin | 3 | 3 | 60.11 | 0.01% | 3 | 93.05 | 0.04% |
| 8.9kDa secreted proteins | 2 | 2 | 1338.30 | 0.19% | 0 | 0.00 | 0.00% |
| 13 kDa | 2 | 0 | 0.00 | 0.00% | 2 | 813.84 | 0.32% |
| 7Cys domain | 2 | 2 | 3013.64 | 0.44% | 0 | 0.00 | 0.00% |
| 17kDa secreted proteins | 1 | 1 | 392.31 | 0.06% | 0 | 0.00 | 0.00% |
| 5.3 kDa | 1 | 1 | 5.65 | 0.00% | 0 | 0.00 | 0.00% |
| Ectonucleotide pyrophosphatase/ phosphodiesterase | 1 | 1 | 1.91 | 0.00% | 1 | 205.04 | 0.08% |
| Cysteine rich secreted Protein | 1 | 1 | 0.61 | 0.00% | 1 | 27.37 | 0.01% |
| P-selectin like | 1 | 1 | 94.70 | 0.01% | 1 | 2.78 | 0.00% |
| Evasin-immunity tick binding chemokine | 1 | 0 | 0.00 | 0.00% | 1 | 15.29 | 0.01% |
| **Enzymes** |  |  |  |  |  |  |  |
| Metalloproteases | 32 | 31 | 27990.75 | 4.04% | 10 | 726.93 | 0.28% |
| Serine Proteases | 19 | 14 | 296.77 | 0.04% | 18 | 9287.33 | 3.60% |
| Lipases | 14 | 13 | 2980.05 | 0.43% | 6 | 683.93 | 0.26% |
| Nucleases | 13 | 11 | 1123.39 | 0.16% | 9 | 939.51 | 0.36% |
| Chitinases | 10 | 8 | 235.70 | 0.03% | 8 | 1053.99 | 0.41% |
| Cathepsins | 7 | 7 | 331.10 | 0.05% | 7 | 7563.16 | 2.93% |
| Serine Carboxipeptidase | 4 | 4 | 194.36 | 0.03% | 4 | 1188.81 | 0.46% |
| Carboxipeptidases | 3 | 3 | 51.72 | 0.01% | 3 | 189.88 | 0.07% |
| Cysteine Proteases | 3 | 3 | 9.24 | 0.00% | 3 | 5621.27 | 2.18% |
| Proteases | 1 | 1 | 15.53 | 0.00% | 1 | 24.07 | 0.01% |
| **Protease Inhibitors** |  |  |  |  |  |  |  |
| Serine proteinase inhibitors | 25 | 21 | 345.97 | 0.05% | 23 | 8095.79 | 3.14% |
| Kazal | 24 | 19 | 21816.59 | 3.15% | 15 | 3096.50 | 1.20% |
| Kunitz domain | 24 | 19 | 21813.75 | 3.15% | 13 | 3420.84 | 1.33% |
| Cystatins | 23 | 17 | 16239.82 | 2.35% | 17 | 22881.01 | 8.87% |
| TIL domain | 4 | 4 | 70.69 | 0.01% | 0 | 0.00 | 0.00% |
| Thyropin | 2 | 1 | 585.95 | 0.08% | 2 | 5052.69 | 1.96% |
| **Hypothetical secreted proteins** | 497 | 385 | 86426.29 | 12.48% | 359 | 119231.83 | 46.20% |
| **Total Secreted** | **886** | **717** | **692248.44** | **100.00%** | **596** | **258101.68** | **100.00%** |
|  |  |  |  |  |  |  |  |

**Supplementary Table 4. Immunity proteins**

| **Functional family** | **Contigs** | **Contigs SG** | **TPM SG** | **%SG** | **Contigs MG** | **TPM MG** | **% MG** |
| --- | --- | --- | --- | --- | --- | --- | --- |
| Cytokine-associated | 48 | 38 | 1004.60 | 9.17% | 45 | 11979.16 | 7.45% |
| Other immunity related | 21 | 20 | 257.42 | 2.35% | 21 | 735.40 | 0.46% |
| Glycoproteins - pattern recognition | 13 | 12 | 298.69 | 2.73% | 13 | 1353.30 | 0.84% |
| Ficolin/Ixoderin | 8 | 7 | 1538.63 | 14.04% | 5 | 751.92 | 0.47% |
| Toll-associated | 8 | 8 | 35.22 | 0.32% | 7 | 93.02 | 0.06% |
| Defensins | 7 | 3 | 17.80 | 0.16% | 7 | 125576.25 | 78.07% |
| ML domain Proteins | 6 | 3 | 5817.41 | 53.10% | 6 | 1180.53 | 0.73% |
| Phospatases | 5 | 5 | 142.46 | 1.30% | 5 | 137.45 | 0.09% |
| Lysozyme | 3 | 3 | 62.14 | 0.57% | 2 | 15405.73 | 9.58% |
| Gamma-interferon inducible lysosomal thiol reductase | 3 | 3 | 677.71 | 6.19% | 1 | 301.77 | 0.19% |
| C-type lectin-like | 2 | 2 | 5.28 | 0.05% | 2 | 182.75 | 0.11% |
| Peptidoglycan recognition protein | 1 | 1 | 15.92 | 0.15% | 1 | 67.80 | 0.04% |
| Microplusins | 1 | 1 | 1078.53 | 9.85% | 1 | 3087.67 | 1.92% |
| Lipocalin receptor | 1 | 1 | 3.22 | 0.03% | 1 | 3.43 | 0.00% |
| **Total Immunity** | **127** | **107** | **10955.03** | **100.00%** | **117** | **160856.18** | **100.00%** |
|  |  |  |  |  |  |  |  |

**Supplementary Figure I. Evolutionary relationships *Ornithodoros rostratus* proteins from the 7DB-family.**The evolutionary history was inferred using the Neighbor-Joining method. The optimal tree with the sum of branch length = 12.96 is shown. The percentage of replicate trees in which the associated taxa clustered together in the bootstrap test (10000 replicates) are shown next to the branches. The evolutionary distances were computed using the Poisson correction method and are in the units of the number of amino acid substitutions per site. The analysis involved 26 amino acid sequences. All ambiguous positions were removed for each sequence pair. There were a total of 727 positions in the final dataset. Evolutionary analyses were conducted in MEGA7.
